# Post-traumatic stress disorder and its associated factors among survivors of 2015 earthquake in Nepal

**DOI:** 10.1186/s12888-023-04836-3

**Published:** 2023-05-15

**Authors:** Radha Acharya Pandey, Pratibha Chalise, Sunita Khadka, Bina Chaulagain, Binu Maharjan, Jyotsna Pandey, Jyoti Nepal, Chandranshu Pandey

**Affiliations:** Department of Nursing, Kathmandu University School of Medical Sciences, Dhulikhel Hospital, Kathmandu University Hospital, GPO Box 11008, Dhulikhel, Kavre Nepal

**Keywords:** Post Traumatic Stress Disorder, Earthquake Survivors, Nepal Earthquake

## Abstract

**Background:**

Natural disasters cause long term psychological consequences, especially post-traumatic stress disorders. It has been regarded as the most prevalent of psychiatric disorders after a natural disaster. The purpose of this study is to estimate the prevalence of Post-Traumatic Stress Disorder (PTSD) and determine its associated factors in adult survivors three years after the 2015 Nepal earthquake.

**Methods:**

A cross-sectional descriptive design was used where 1076 adults within the age range of 19–65 were randomly selected and interviewed from four adversely affected districts due to the 2015 earthquake. Instruments included a demographic questionnaire, an earthquake exposure questionnaire, the Oslo Social Support Scale (OSSS), and the Post-traumatic Stress Disorder Checklist-Civilian Version (PCL-C). Descriptive and inferential statistics were applied using Statistical Package for Social Science (SPSS) Version 16 for data analysis.

**Results:**

The prevalence of PTSD among earthquake survivors was 18.9%. The multivariate logistic regression showed that gender, ethnicity, education, occupation, social support and severity of damage to house and property were significantly associated with PTSD. Odds of having PTSD was 1.6 times higher among females (AOR = 1.6, 95% CI: 1.1–2.3) and nearly 2 times higher amongst illiterate survivors (AOR = 1.9, 95% CI: 1.2–2.8). Participants from the Janajati ethnic group and those who had a business occupation had a 50% lower risk of having PTSD. Around 39% of the participants had moderate social support and had 60% lower odds of having PTSD compared to those with poor social support (AOR = 0.4, 95%CI: 0.2–0.5, *p* < 0.001). Participants with medium and very high-level damage to personal property were more likely to have PTSD.

**Conclusion:**

Post-traumatic stress disorder remained prevalent amongst survivors three years after the 2015 Nepal Earthquake. It is important to provide psychological and social support for survivors to decrease the health burden from PTSD. Socio-demographic characteristics such as females, farmers, those survivors who endured significant personal property damage were at higher risk.

**Supplementary Information:**

The online version contains supplementary material available at 10.1186/s12888-023-04836-3.

## Background

Nepal was hit by a devastating 7.8 magnitude earthquake with its epicenter in Barpak, Gorkha District, northwest of Kathmandu, at 11:55 a.m on April 25, 2015 (Nepal Standard Time). 17 days later, on May 12, 2015, another 6.8 magnitude aftershock caused further damage and suffering [1]. These two large earthquakes resulted in considerable property damage. In several districts, people's lives and infrastructure were in jeopardy [2]. The earthquake caused 8,896 fatalities and 22,303 seriously injured patients. More than 600,000 households were fully damaged leaving 300,000 partially damaged. Of these households, it is estimated that 450,000 people remained homeless [1, 3]. According to the World Health Organization (WHO), 20% of people in post-earthquake and other humanitarian situations experience psychological distress, with a lower minority (3–4%) experiencing severe mental disorders [4].

Earthquakes are one of the world’s major calamities. With very little warning, it destroys personal property and lives [5]. It may affect people physically as well as mentally, causing them to be more afraid of the earthquake due to its unpredictable and unforeseen nature [6].These experiences can be traumatic especially to the children, women and elderly [5]. Physical injuries can be treatable, as it can be viewed externally, but mental injury is often ignored as it is not visible [5].

More than two-thirds of the world's populations have experienced an earthquake at some point in their lives, resulting in a wide range of mental and physical health consequences [2]. Approximately two-thirds of the PTSD patients may improve over time, while for others, it may continue to damage their cognitive and behavioral functioning for years. As a result, it is necessary to give ancillary resources and social support in the aftermath of an earthquake to assist individuals in coping with stressful effects of an earthquake [5].

Numerous previous studies outside of Nepal have shown the prevalence rate of depression after earthquakes ranging from 16 to 28 percent, while PTSD prevalence rates range from 7 to 40 percent [7–9]. Similarly, a study done in China (Wenchuan, 2008) found that the prevalence of PTSD in earthquake survivors varied from 10.3 to 66%. Prevalence correlated with the level of trauma exposure and proximity to the epicenter of the earthquake [10]. Another study one year after the 2008 Wenchuan earthquake showed that the prevalence of PTSD was 40.1% in Wenchuan, China, over the four-year period following its 2008 [8]. According to a systematic review of mental health disorders following the Great East Japan Earthquake in 2011, the reported prevalence of PTSD ranged from 10 to 53.5%, while the prevalence of depression ranged from 3.0 to 43.7% [11].

Previous mental health research in Nepal has focused on heightened rates of psychological distress among a substantial proportion of the population affected by the 2015 Nepal earthquakes, with a smaller proportion reporting more severe mental health and psychosocial difficulties [3]. Research conducted in 2015 Dhadingdistrict earthquake survivors showed the prevalence of PTSD at 18.5% [5]. Another study amongstadolescents in Sindhupalchok and Kathmandu districts showed a PTSD prevalence of 39.5% and 10.7% three years after the earthquake [12]. The literature review retrieved PTSD measures immediately after earthquakes at 4, 9, 10 and 14 months in Nepal, concluding that PTSD remains for a long time [2, 5, 13, 14].

According to cross-sectional community-based research, 27.5 percent to 33.7 percent of individuals passed the Beck Depression Inventory criterion for depression, 22.9 percent to 27.7 percent for anxiety and 9.6 percent for PTSD [15, 16]. According to longitudinal follow-up, the frequency was higher among women and older age groups, as well as following armed conflict [16, 17].

In a quantitative survey of 513 community members, those with symptom scores suggesting depression (34.2 percent), anxiety (33.8 percent), and alcohol-use issues (20.4%) were in greater proportions than those with PTSD symptoms (5.2 percent) [3]. In the four months following the earthquake, the prevalence of suicidal ideation was alarmingly high, at 10.9 percent [3, 18].

According to a study by S. Sherchan et al., following the earthquake, mental health services were offered in the form of hospital-based clinical services, mobile health camps, and health worker training workshops to deliver basic mental health services [19].

Despite the fact that long-term psychological consequences of disasters are significant and common, little attention has been paid to the long-term psychological consequences of disasters [20]. More research on the long-term psychosocial consequences of disasters is neededespecially in Nepal as the country is at risk of major earthquakes in the future [21, 22]. To our knowledge, little research has been conducted in this area 3 years after the Nepal earthquake. Few studies have researched prevalence or quality of life in individuals with trauma/PTSD [23]. A systematic review revealed that most of the research was done 1 to 20 months after the 2015 earthquake in Nepal in limited earthquake affected areas only [24]. The finding of PTSD was mostly present among the earthquake survivors with rates varying from 4.9% to 51%. Gender and age were associated factors for this study. However, earthquake related variables weren't observed in this study [24]. Therefore, the goal of this study is to fill a gap in the literature by looking at the prevalence of PTSD and associated socio-demographic factors in adult survivors who are living in the most affected areas beyond 3 years after the 2015 earthquake. The identification of variables that are of influence in the 2015 earthquake in Nepal can educate and assist researchers in future exploration of the topic.

## Methods

### Study design, site and study population

The research was a descriptive cross-sectional study conducted in four earthquake-affected districts (Gorkha, Dolakha, Sindhupalchowk and Bhaktapur) in 2019, three years after the Nepal earthquake. The study population were young, middle and older adults who were the survivors of the devastating earthquake. Data was collected from 1076 randomly selected adults from the four adversely-affected districts. Participants aged between 19 and 65 years, who had experienced the earthquake, 2015 in Nepal and spoke Nepali were included in the study. Participants who had hearing problems, poor verbal communication, severe disease conditions, who were already identified or recorded in a health facility as intellectually disabled, dementia or any other major psychosis, and severe mental disorders under medication were excluded from the study.

### Sampling strategy

**Fig. 1:**
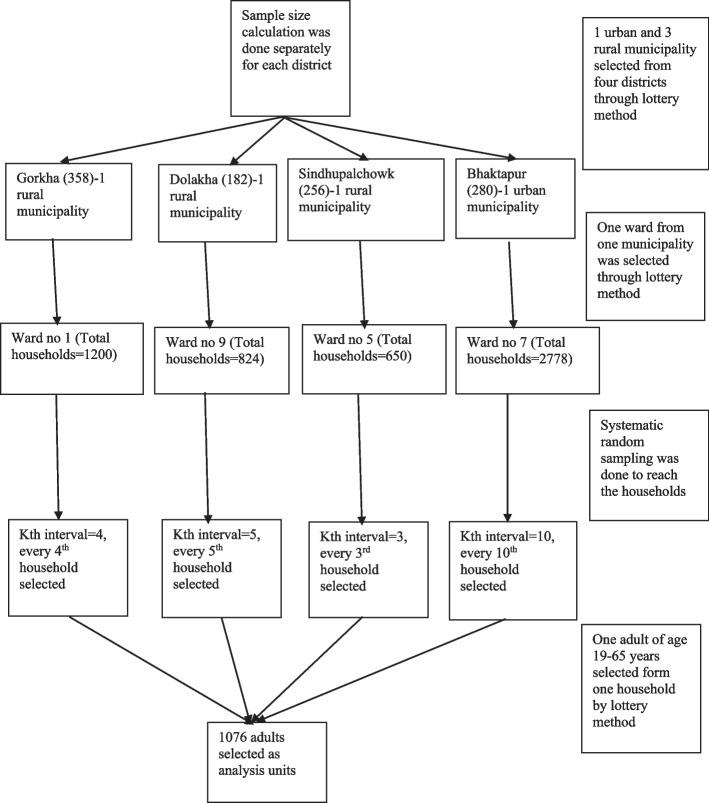
Flowchart of sampling strategy

Sample size calculation was done (Fig. 1) for the four most earthquake affected districts (Gorkha, Dolakha, Sindhupalchowk and Bhaktapur). The sample size from Gorkha was 358, 182 from Dolakha, 256 from Sindhupalchowk and 280 from Bhaktapur to make the final sample size of 1076.

Multi-stage random sampling was done. At the first stage (firstly), three rural municipalities (Gorkha, Dolakha, Sindhupalchowk) and one urban municipality (Bhaktapur) were selected randomly. The list of the total number of administrative wards in each of the four municipalities and the total number of houses destroyed in each of the wards was obtained from the ward offices. The household destruction percentage of each ward was calculated. On the basis of the household destruction percentage, the wards with over 50 percent household destruction were considered as the most affected. Secondly, one of the most affected wards was selected through simple random sampling (lottery method) from each four municipalities. Thirdly, systematic random sampling was done to select households. Due to the earthquake and internal migration, it was difficult to determine the population size and the list of people (age 19–65 years) in selected wards.

The total number of households and the sampling frame (list of households) in the selected wards were obtained from respective ward offices. The total number of present households in each ward was divided by the required sample size of the respective ward to determine the different kth intervals for each of the four wards.

The first household was selected through a lottery method. Then, every kth household was selected for the study from the list of households in each ward accordingly until the required sample size was met. Only one participant of age 19 to 65 years within a household was recruited for interview through lottery method to ensure independence of study subjects.

### Ethical considerations

This study was approved by the Institutional Review Committee of Kathmandu University School of Medical Sciences at Dhulikhel, Nepal. All participants were informed about the purpose and procedures of the study. Participation in the study was voluntary. They were allowed to withdraw their consent and discontinue the research interview at any time. Before the interview began, written informed consent was taken from the participants. Anonymity and privacy of the participants were maintained during data collection.

### Data collection tools

Face to face interview was conducted using a structured questionnaire. Before data collection, four enumerators with Bachelor’s degree in nursing were called together to review the questionnaire and each item was explained. They were also trained for face-to-face interview methods. The first part of the questionnaire included socio-demographic questionnaire such as age, gender, marital status, ethnicity, religion, occupation and literacy status. The second part included a questionnaire about earthquake exposure such as self-injury, loss of family or friends, loss of house and property, witness of burial, etc. Earthquake exposure was considered “Exposed” when participants answered “Yes” to any of these earthquake exposure related questionnaires.

In the third part, post-traumatic stress symptoms were assessed using the subscale of PTSD Checklist-Civilian Version (PCL-C) to assess PTSD prevalence. The PCL-C version is a commonly used instrument to assess PTSD. It has 17 items which measures three clusters of symptoms that record into diagnostic Criteria B (re-experiencing), C (avoidance/ numbing), and D (hyperarousal) for PTSD in the Diagnostic statistical manual for mental health (DSM-IV) was adopted (Ni et al., 2013) [25, 26]. This tool allows the participant to indicate the extent to which each symptom is correlated with the earthquake on a scale from 1 (not at all) to 5 (extremely). The Nepali translated version of the PCL-C was administered for measuring PTSD. The value of Cronbach alpha in this study for PCL-C was found to be 0.95, which possesses excellent internal consistency. All three clusters of symptoms were found to be acceptable in this study setting. The area under the curve for B, C and D groups of symptoms were 0.97, 0.98, and 0.96 respectively, which shows the tool is acceptable in Nepalese contests [5]. A provisional PTSD diagnosis can be made by adding up all items for a total severity score or treat response categories 3–5 (moderately or above) as symptomatic and responses 1–2 (below moderately) as non-symptomatic, then the DSM criteria is used for its diagnosis. The maximal total score for 17 items is 85. Participants with a score of 50 or greater were classified as having probable PTSD. With a PCL-C score of 50 as the diagnostic cut-off, the sensitivity was approximately 80% [23, 27–34]. According to a study done in Nepal, the value of Cronbach alpha of PCL-C was found to be 0.95, which possesses excellent internal consistency [5]. The PCL-C was previously validated in Nepal with total scores of 50 or above indicative of probable PTSD (Thapa&Hauff, 2005, Dhungana, 2021) [3, 23, 35]. Besides Nepal, a study done in 3372 pregnant women in Peru revealed the reliability of the PCL-C was excellent (α = 0.90). Receiver operating characteristics analysis showed that a cut-off score of 26 offered optimal discriminatory power, with sensitivity of 0.86 (95% CI: 0.78 – 0.92) and a specificity was found of 0.63 (95% CI: 0.62 – 0.65) [36].

The social support scale was the fourth section of the questionnaire. The Oslo 3-items Social Support Scale (OSSS-3) is one of the best indicators of mental health since it is a brief measure of social functioning. It assesses the number of persons with whom the respondent feels close, as well as the level of attention and care provided by others and the ease with which they can seek practical assistance. The Oslo Social Support scale scores ranged from 3–14, with 3–8 indicating low (poor) support, 9–11 indicating moderate support, and 12–14 indicating strong support [37]. Internal consistency for the Oslo Social Support Scale was found to be good with the Cronbach alpha of 0.82, which was also validated in Nepal [5].

Finally, data was collected from 1076 total adult survivors (one in each house) selected from four different districts.

### Data analysis

All data were analyzed by statistical software SPSS 16.0. Descriptive analysis was done to report frequency, percentage, mean and standard deviation. Chi-square test was done to find association between PTSD and sociodemographic variables, earthquake exposure variables and social support. Binary logistic regression was used to determine the relationship between PTSD and socio-demographic variables, earthquake exposure variables and social support.

*P* value of < 0.05 was considered to be statistically significant.

## Results

Table 1 depicts the distribution of socio-demographic variables, social support and their association with PTSD. The prevalence of PTSD was 18.9%. The socio demographic variables were associated with PTSD such as age (*p* = 0.003), gender (*p* = 0.001), ethnicity (*p* < 0.001), education (*p* < 0.001), marital status (*p* = 0.010) and occupation (*p* < 0.001). Social support was also associated with PTSD (*p* < 0.001).

**Table 1:** Socio-demographic characteristics and social support of participants (*n* = 1076)

**Characteristics**		**No PTSD** **n (%)**	**PTSD** **n (%)**	***p*** **-value**
	**Total n(%)**	873(81.1)	203(18.9)	
**Age** Mean (SD) = 40.48(13.43)				
Young and Middle adult	951(88.4)	784(82.4)	167(17.6)	0.003*
Old Adult	125(11.6)	89(71.2)	36(28.8)	
**Gender**				
Male	470(43.7)	402(85.5)	68(14.5)	0.001*
Female	606(56.3)	471(77.7)	135(22.3)	
**Ethnicity**				
Brahmin/Chhetri	350(32.5)	259(74)	91(26)	< 0.001*
Janajati	637(59.2)	543(85.2)	94(14.8)	
Dalit	89(8.3)	71(79.8)	18(20.2)	
**Religion**				
Hindu	811(75.4)	664(81.9)	147(18.1)	0.503
Buddhist	188(17.5)	147(78.2)	41(21.8)	
Christian and others	77(8.1)	62(80.5)	15(19.5)	
**Educational status**				
Literate	857(79.6)	725(84.6)	132(15.4)	< 0.001*
Illiterate	219(20.4)	148(67.6)	71(32.4)	
**Marital status**				
Married	885(82.3)	706(79.8)	179(20.2)	0.010*
Unmarried	165(15.3)	147(89.1)	18(10.9)	
Widow	26(2.4)	20(76.9)	6(23.1)	
**Occupation**				
Agriculture	535(49.7)	396(74)	139(26)	< 0.001*
Business	163(15.1)	146(89.6)	17(10.4)	
Service	101(9.4)	91(90.1)	10(9.9)	
Home Makers	136(12.6)	120(85.1)	16(11.8)	
Others (Students, Labor)	141(13.2)	120(85.1)	21(14.9)	
**Social support**				< 0.001*
Poor support	499(46.4)	368(73.7)	131(26.3)	
Moderate support	417(38.8)	376(90.2)	41(9.8)	
Strong support	160(14.9)	129(80.6)	31(19.4)	

^*^Statistically significant (*p* < 0.05) at 95% CI (Confidence Interval)

In the study, most of the participants (88.4%) were young and middle adults where 17.6% of them had PTSD and 28.8% of older adults had PTSD. Again, more than half of the participants (56.3%) were females where 22.3% of females and 14.5% of males had PTSD. More than half of the participants (59.2) belonged to the Janajati ethnic group where 14.8% of them had PTSD. Majority (75.4%) of participants were Hindu and 18% of them had PTSD. Likewise, majority (79.6%) were literate and only 15% of them had PTSD. Most of the participants (82.3%) were married and among them 20% had PTSD. Agriculture was the most common (49.7%) occupation among the participants where 26% of them had PTSD.

Regarding social support scale, nearly half of the participants (46%) had poor social support. Among participants with poor social support, 26.3% of them had PTSD. In participants who had moderate social support, only 9.8% had PTSD and in people with strong social support, 19% had PTSD.

### Earthquake exposure of the participants

Majority (87%) of the respondents were exposed to the earthquake characteristics. Regarding earthquake exposure of the participants, most of the exposure variables were associated with PTSD such as self-buried (*p* = 0.006), self-injured (*p* < 0.001), witness of injured in earthquake (*p* = 0.001), witness of buried in earthquake (*p* = 0.001), witness of death in earthquake (*p* = 0.002), family members being handicapped (*p* = 0.005), death of family members (*p* = 0.012), loss of house and property (*p* < 0.001) and severity of damage to house and property (*p* < 0.001). Other exposure variables such as self-handicapped and relatives and friends being handicapped were not associated with PTSD.

Majority of the participants (92.4%) had lost house or property in the earthquake. Among them, 20% had PTSD. Among the 82 participants who were self-buried, 30.5% had PTSD. Twenty two percent of the participants who had witnessed death in earthquake had PTSD. Again, among the participants who had death in their family, 26% of them had PTSD. The estimates related to earthquake exposure are not shown in tables.

Table 2 illustrates the relationship between PTSD and several associated factors. The multivariate logistic regression shows that gender, ethnicity, education, occupation, social support and severity of damage to house and property are significantly associated with PTSD. Odds of having PTSD is 1.6 times higher among females compared to males (AOR = 1.6, 95% CI: 1.1–2.3, *p* = 0.008). The people in Janajati ethnicity have 40% lower odds of having PTSD compared to Brahmin/Chhetri (AOR = 0.6, 95%CI: 0.4–0.9; *p* = 0.017). Participants who were illiterate are nearly 2 times more likely to have PTSD compared to literate (AOR = 1.9, 95%CI: 1.3–2.9, *p* = 0.001). Participants who did business had 60% lower risk of having PTSD compared to farmers (AOR = 0.4, 95% CI: 0.2–0.9, *p* = 0.028). The participants with moderate social support had 70% lower odds of having PTSD compared to those with poor social support (AOR = 0.3, 95%CI: 0.2–0.5, *p* < 0.001). Participants who had medium to very high level damage to house and property were nearly 5 times more likely to have PTSD compared to those who did not have any damage to house and property or very less damage (AOR = 4.9, 95%CI:2.3–10.5, *p* < 0.001).

**Table 2:** Post-Traumatic Stress Disorder (PTSD) and its associated factors (*n* = 1076)

Characteristics	Univariate			Multivariate		
	**COR**	**95% CI**	***p*-value**	**AOR**	**95% CI**	***p*-value**
**Age**						
Young and Middle adult	ref					
Old Adult	1.9	1.2–2.9	0.003*	1.2	0.8–2.1	0.372
**Gender**						
Male	ref					
Female	1.7	1.2–2.3	0.001*	1.6	1.1–2.3	0.008*
**Ethnicity**						
Brahmin/Chhetri	ref					
Janajati	0.4	0.3–0.7	< 0.001*	0.6	0.4–0.9	0.017*
Dalit	0.7	0.4–1.3	0.262	0.8	0.4–1.4	0.366
**Religion**						
Hindu	ref					
Buddhist	1.2	0.8–1.8	0.245	1.5	0.9–2.3	0.061
Christian	1.1	0.6–2.1	0.570	0.9	0.5–1.8	0.885
**Educational status**						
Literate	ref					
Illiterate	2.6	1.8–3.7	< 0.001*	1.9	1.3–2.9	0.001*
**Marital status**						
Married	ref					
Unmarried	0.5	0.3–0.8	0.006*	0.6	0.3–1.1	0.116
Widow	1.2	0.5–3.0	0.722	1.1	0.4–3.2	0.820
**Occupation**						
Agriculture	ref					
Business	0.3	0.2–0.5	< 0.001*	0.4	0.2–0.9	0.028*
Service	0.4	0.2–0.6	0.001*	0.7	0.3–1.2	0.176
Home Makers	0.3	0.2–0.7	0.001*	0.5	0.2–1.0	0.062
Others(Students, Labour)	0.4	0.3–0.8	0.013*	0.7	0.4–1.3	0.395
**Social Support**						
Poor support	ref					
Moderate support	0.3	0.2–0.4	< 0.001*	0.3	0.2–0.5	< 0.001*
Strong support	0.7	0.4–1.0	0.080	0.7	0.4–1.2	0.219
**Earthquake exposure**						
Not exposed	ref					
Exposed	0.9	0.5–1.4	0.713	1.5	0.8–2.5	0.118
**Severity of damage to house and property**						
No to Very less	ref					
Medium to Very high	5.8	2.8–12.1	< 0.001*	4.9	2.3–10.5	< 0.001*

*COR* Crude Odds Ratio, *AOR* Adjusted Odds Ratio, *Ref* Reference category

^*^Statistically significant (*p* < 0.05) at 95% CI (Confidence Interval)

## Discussion

Earthquakes cause significant stress and affect a substantial number of people in the world. The 2015 earthquake in Nepal was a dreadful disaster with a painful impact on many survivors. Three years after the disaster, affected people have yet to fully recover. This cross-sectional study emerged within the four selected most affected districts. The PTSD prevalence assessed using PCL-C (DSM-IV) criteria was remarkably high three years after the disaster.

### Prevalence of the PTSD

The present study found that the prevalence of PTSD three years after a devastating earthquake is 18.9% among earthquake-affected adult survivors, which is consistent with previous studies done in Nepal on mental health problems nine months after the 2015 Nepal earthquake [5].

A study containing forty-six eligible articles showed the combined incidence of PTSD among survivors who were diagnosed, at no more than nine months after the earthquake, was 28.76%, while for survivors who were diagnosed at over nine months after earthquake the combined incidence was 19.48% [38]. This result shows the prevalence of PTSD remains high years later. A study on 1355 adults from the 2010 Haiti earthquake after 30 months (about 2 and half years) found the prevalence rates of PTSD was 36.75%, which is higher than our study [39]. The PTSD was measured by the clinician using life events checklist subscale [39].

Similarly, our prevalence rates are lower than the 40% rate three years after the Turkey earthquake. The epicenter was predominantly government-constructed housing. The study used the Traumatic Stress Symptom Checklist (TSSC), which included 17 DSM IV PTSD, 55.2% female, 33.4% male reported eighteen months after the Kashmir earthquake those people were living in tents, 28.5% reported 11 months after the 2015 Nepal earthquake, 23% reported 14 months after the epicenter of Turkey earthquake, 22.1% reported 8 months after the 2008 Wenchuan earthquake in China [10, 40–43].

Most long-term studies of prevalence of PTSD in disaster exposed survivors show that the prevalence of PTSD decreases with time [44]. In contrast, some studies show that there might be a rise in the prevalence of PTSD. In the years following exposure to the 2001 World Trade Center disaster, the prevalence of PTSD among firefighters was shown to increase over time, from 9.8% in the first year (2002) to 10.6% in the 4^th^ year (2005) (*p* = 0.0001), using the same scale as ours [45].

A literature review shows that most researchers who studied PTSD rates occurred around three months after the earthquake [10]. Even after powerful quakes (Wenchuan), the prevalence rate of PTSD was seen lower than other reports in the short term, however, our study focused on the potential long-term effects after an earthquake. These differences may be explained by factors such as the loss of family, loss of property, loss of job, self-injury/buried, gender, strength of coping, type of exposure, location of the disaster and diagnosis measure (tools) of PTSD. Compared with current study results of Nuwakot (2016) the prevalence (24.10%) was found to be higher in the Nepal earthquake after 10 months, it might be because of the short duration after an earthquake than ours [2]. This is also supported by a study done in China after 9 month of an earthquake which found PTSD 24.2% by using DSM IV criteria [46].

A study done in the Sichuan and Shaanxi provinces of China, 2 earthquake-stricken areas, showed that the 1 –year prevalence of PTSD amongst 2080 adult survivors was 40.1% [8]. Three years after the same earthquake it was found that 10.3% of the respondents had PTSD [47]. Similarly, a population survey done among 1756 respondents aged 16–98 in the 1991 earthquake in Yu-Chi, Taiwan found that at 6 months and 3 years, the estimated rate of Post-traumatic Stress Symptoms (PTSS) was 23.8% and 4.4% respectively [48]. Likewise, another study (Turkey) showed PTSD prevalence dropped by the length of time after the earthquake [43]. However, some other studies suggest the prevalence of PTSD has not decreased as time goes on [42, 47, 49].

After an extensive literature review and comparing our results we conclude that many factors contribute to the prevalence of PTSD symptoms amongst earthquake survivors. Further comparison is limited due to lack of studies measuring the prevalence of PTSD among survivors three years after the earthquake in Nepal. We can also assume that variation may exist between magnitude of earthquake, assessment tools, type of study (qualitative, quantitative) and coping strategies adopted by individuals after a traumatic event.

In our study, there was a significant association (*p* = 0.003) with age group and PTSD. In the age group of 60 and above, participants had the highest prevalence of PTSD (28.8%). These findings were consistent with a study done in Dhading Nepal, 9 months after the earthquake where adolescents and the elderly were associated with a higher incidence of PTSD [5, 50]. Similarly, another study done in Nepal (2019) and other countries (China, 2011, 2013 and 2014, Tehran, 2017), revealed that the elderly were more likely to have PTSD compared to younger adult survivors [2, 8, 51–54]. Our result is also consistent with a study done by Bhat W. et al. where age was one of the best predictors of PTSD prevalence [55].

Although our study is consistent with most other studies, it was inconsistent with the findings done in Wenchuan one month after the earthquake, which revealed the prevalence of PTSD as in young and middle age adult was 78.8% [56]. One probable reason is the ‘burden perspective hypotheses’, which suggests that middle-aged adults experience poorer coping capacity than others because of their responsibilities to society (e.g. working) and to the family (e.g. often providing support to both children and parents), which can render them more psychologically vulnerable in the aftermath of the disaster [57, 58]. Older adults have a high coping mechanism according to socio-cultural structure (potential spiritual, religious beliefs), which is consistent with a study done by Palgi. Y. [59, 60]. Moreover, older people were reported to learn coping mechanisms from experience; also, older age was associated with less sensitivity, fewer negative beliefs, and decreased mood symptoms [61].

Gender difference in prevalence of PTSD was also noted in our study. Females were found to have a 1.6 times higher odds ratio of developing PTSD compared to males. This is consistent with a study done by Lama et al. [54].

Gender differences are also supported from previous studies done in Nepal (2018) and other countries (China, 2015, Pakistan, 2011, 2013, and China, 2013) [5, 53, 61–66]. A study by Shrestha after the 2015 Nepal Earthquake also revealed higher PTSD amongst female medical personnel [67]. Higher PTSD risk among women may be due to their stronger perceptions of threat and loss of control [2]. In addition, it is possible that women are more susceptible to negative events and tend to express their emotions more than males [5]. Literature even explains gender differences in neuro-endocrine response that may lead to higher risk of PTSD in women [68].

In our study, illiterate survivors were nearly 2 times more likely to have PTSD compared to literate survivors. This is consistent with a study in Wenchuan (2012), where PTSD was found to be 52.6% among respondents with a low level of education (primary and secondary) compared with a university degree (master's and bachelor's degree). Another survey result revealed that in Nepal, the prevalence of PTSD was 13.9% among literate survivors, while illiterate accounted for 38.1% [2, 50]. A lower education level may contribute to patients being less informedwhich may decrease confidence in both physical and mental recovery [2].

The results of the present study differs from a study conducted in Haiti and other countries (China, Pakistan) which showed that higher education increases levels of PTSD [39, 62, 65]. Our study also disagrees with studies by Contractor, AA and Khodadadi-H. et al. which found that participants with a high school education were more likely to have PTSD [69]. Their hypothesis for these results is that due to their increased level of education they may be moreinclined to worry about future consequences regarding earthquake than illiterate respondents.

Our study found that ethnicity also had an association with PTSD symptoms. In the current literature, ethnicity has not been consistently described as a risk factor associated with PTSD [37, 68].

In our study, the profession of respondents was also found to have an association with PTSD. This is supported by a study conducted in China 5 years after the earthquake in 2013 [62]. The major occupation of the people residing in Wenchuan, China was fish farming (aquaculture). It was impacted or destroyed by the 2008 earthquake, and, as a result, their source of income was affected [62]. Similarly, a study revealed psychological health disturbances were more common in farmers and farmworkers, conducted by Daghagh Yazd S et al. [70].

People engaged in other occupations where less likely to develop PTSD when compared with farmers or those engaged in agriculture. Our result was consistent with a study done by Mitsuaki Katayanagi et al. which revealed that after the Great East Japan Earthquake in 2011 nearly half of the farmers who were engaged in agriculture and fishery reported decreased income [71]. It is possible that these survivors had fewer resources in coping with the stress from the economic burden than those of other occupations [62]. This may resulted in a higher PTSD rate amongst farmers [62].

In our study perceived social support was associated with PTSD in both univariate and multivariate analysis (*p* < 0.001). In this study, participants with moderate social support had 70% lower odds of having PTSD compared to those with poor social support (AOR = 0.3, 95%CI: 0.2–0.5, *p* < 0.001). These findings are similar to findings by Asnakew S. et al. who found that participants with poor social support were 3.6 times more likely to develop PTSD than those with strong social support (AOR = 3.6, 95% CI from 2.0 to 6.7) [72]. Other research has shown that there is a possible positive effect of social support for the prevention of PTSD. Social support will not benefit everyone equally. Numerous confounding variables, including gender, previous experiences of violent trauma, and self-efficacy, appear to play a role [73]. Another study by Dai W et al in 2016 found that strong social support can not only protect individuals from mental disorders but also facilitate psychological recovery from disaster [38]. This variation could be due to differences in the study population, assessment methods and posttraumatic period.

### Earthquake related variables and PTSD related information

This study showed that there is a significant association between PTSD prevalence and factors such as getting buried after an earthquake, death of a family member, and witnessing others who have been buried, injured ordied. This finding is consistent with other previous studies [39, 74].These are all autonomous factors for developing PTSD. Similarly, survivors with family member loss may undertake more negative traumaand endure greater economic and psychological pressure,which may contribute to a higher PTSD prevalence rate [75].

Another factor that was associated with the PTSD prevalence was participant house and property damage. Those who had medium to very high level damage to house and property were 5 times more likely to have PTSD compared to those with no or very less damage. A study in Dhadhing showed participants who had house completely damaged and loss of property had between 4 and 9 times higher odds of having PTSD [5].

Our study is consistent with numerous previous studies [5, 7, 8, 62, 76, 77]. It is a common association that increases the prevalence rate of PTSD and likely was confounded by the fact that none of the respondents were living in their previous houses three years after the earthquake. Their current way of living and surrounding environment may have caused a constant reminder to the patient of the earthquake. The temporary accommodation of the respondents has likely made an impact on the prevalence of PTSD.

According to Galea and colleagues’ studies, loss of private property (e.g. house) and experiencing economic loss (e.g. loss of job) are major stressors after a disaster [44].This is consistent with our study. In our study sample, over ninety percent of houses were damaged during the earthquake. With loss of houses and property, many survivors were faced with financial and social problems/barriers three years later.

Similarly, consistent with our findings, a study (Kun P. et al. 2009 and 2013) of PTSD concluded that female gender, being married, ethnicity, death of family members, and damaged personal property are associated risk factors [53, 64]. Guo J. et al. and Zhou X. et al. revealed that risk factors for PTSD included advanced age, female gender, buried in the earthquake, injured in the earthquake, witnessing someone get injured in the earthquake, witnessing someone get buried in the earthquake, and witnessing someone die in the earthquake. These findings are consistent with our study findings [10, 75].

### Strength of the study

The study included a large sample of communities most affected by the 2015 earthquake in 4 districts of Nepal. A standardized and validated PCL-C scale was used to assess PTSD symptoms. All interviews were conducted by trained researchers. There was, therefore, little missing data.

### Limitations of the study

Our study has some limitations. Our design is a cross-sectional study with data collected at a single time reference point. To better study the effects of time, additional time points would be helpful. This study is quantitative in nature. The findings would be stronger if it had been triangulated by qualitative (study) interviews. Similarly, we also could not assure that all participants properly disclosed their mental illness. Participants were asked if they were suffering from mental illness and/or if they were under medication as exclusion criteria. It is possible that some participants who responded no had an underlying mental illness. We also did not inquire about our respondents’ family income. Family history of psychiatric illness was also not asked.

Ideally, the questionnaire should be completed by the respondents, but many of the respondents were illiterate. Researchers had to verbally ask questions and record their responses. This may have introduced bias into the study. Although the self-administration of the questionnaire would be more preferable, our data collector might have faced difficulties in explaining the PCL-C questionnaire during data collection due to its difficulty in comprehension. Recall bias may have also been a limitation. Although widely used around the world, PCL-C is a screening measure for PTSD, not a diagnostic tool. The current study participants with a score of 50 or above greater were classified as having probable PTSD without a clinical interview.

## Conclusions

Our findings conclude those three years after the 2015 earthquake, adverse mental health impact still. PTSD remains a common mental health problem among survivors. Multivariate logistic regression shows that associated risk factors of survivors with persistent PTSD include gender, ethnicity, educational status, occupation, social support and severity to loss of house and property. To reduce psychiatric problems such as PTSD and to improve overall mental health and wellbeing in earthquake-affected communities, a community-based mental health and psychosocial support program should be implemented. We also recommend further mix method studies for triangulation between quantitative and qualitative information.

## Supplementary Information

**Additional file 1.**

## Data Availability

The data is available from the corresponding authors upon reasonable request.

## Abbreviations

CI: Confidence Interval
DSM-IV: Diagnostic and Statistical Manual of Mental Disorders 4th Edition
DSM-5: Diagnostic and Statistical Manual of Mental Disorders 5th Edition
IRCKUSMS: Institutional Review Committee of Kathmandu University School of Medical Sciences
OR: Odds Ratio
OSSS: Oslo Social Support Scale
PCL-C: PTSD (Post Traumatic Stress Disorder) Checklist—Civilian Version
PTSD: Post Traumatic Stress Disorder
PTSS: Post Traumatic Stress Symptoms
SD: Standard deviation
SPSS: Statistical Package for Social Sciences
UGC: University Grants Commission
WHO: World Health organization
